# Lessons Learned and Legacy of the Stop Transmission of Polio Program

**DOI:** 10.1093/infdis/jix163

**Published:** 2017-07-01

**Authors:** Yinka Kerr, Melinda Mailhot, Alford (A. J.) Williams, Virginia Swezy, Linda Quick, Rudolf H. Tangermann, Kirsten Ward, Amalia Benke, Anna Callaghan, Kathleen Clark, Brian Emery, Jessica Nix, Eleanor Aydlotte, Charlotte Newman, Benjamin Nkowane

**Affiliations:** 1 Global Immunization Division, Centers for Disease Control and Prevention, Atlanta, Georgia; and; 2 World Health Organization, Geneva, Switzerland

**Keywords:** olio eradication, capacity building, workforce development.

## Abstract

In 1988, the by the World Health Assembly established the Global Polio Eradication Initiative, which consisted of a partnership among the World Health Organization (WHO), Rotary International, the Centers for Disease Control and Prevention (CDC), and the United Nations Children’s Fund. By 2016, the annual incidence of polio had decreased by >99.9%, compared with 1988, and at the time of writing, only 3 countries in which wild poliovirus circulation has never been interrupted remain: Afghanistan, Nigeria, and Pakistan. A key strategy for polio eradication has been the development of a skilled and deployable workforce to implement eradication activities across the globe. In 1999, the Stop Transmission of Polio (STOP) program was developed and initiated by the CDC, in collaboration with the WHO, to train and mobilize additional human resources to provide technical assistance to polio-endemic countries. STOP has also informed the development of other public health workforce capacity to support polio eradication efforts, including national STOP programs. In addition, the program has diversified to address measles and rubella elimination, data management and quality, and strengthening routine immunization programs. This article describes the STOP program and how it has contributed to polio eradication by building global public health workforce capacity.

## DEVELOPMENT AND IMPLEMENTATION OF THE STOP TRANSMISSION OF POLIO (STOP) PROGRAM

When the STOP program was created in 1999, the target date for achieving global polio eradication was 2000. The fact that polio still remained endemic in 20 countries caused concern among the global partnership that the target date for polio eradication would not be met, as occurred with the smallpox eradication program, which achieved eradication many years after the projected completion date [1]. Possible repercussions of such a failure included the loss of credibility of the polio eradication program, the risk of losing financial support to maintain critical eradication activities, fracturing of the fragile eradication alliance among the United Nations (UN) member states, and damage to the conceptual integrity of eradication science.

Increased clarity was brought to the specific tasks that were required to achieve eradication and to the constraints on countries’ and the global community’s capacity to perform those tasks, including planning at all levels, strengthening subnational acute flaccid paralysis (AFP) surveillance, managing polio mass vaccination campaigns, conducting social mobilization, and instituting effective data management. Although all UN member states agreed to the polio eradication goal, some countries lacked the infrastructure or capacity to fully implement what was needed to achieve the goal, placing increased burden on already limited resources at all healthcare system levels. For the remaining polio-endemic countries, the need for technical assistance in performing these required tasks was clear not only at the national level, but at the subnational level, as well. For example, while national eradication plans were generally adequate, implementation of plans frequently lost support at the peripheral levels of the health system, especially in large countries. Expert technical assistance in support of eradication was primarily provided at the global level and to countries by international agencies (primarily the World Health Organization [WHO], the Centers for Disease Control and Prevention [CDC], the United Nations Children’s Fund [UNICEF], and Rotary International); however, these agencies found it difficult to provide the sustained assistance that was needed.

In 1998, the CDC’s director, Dr Jeffery Koplan, called a meeting between staff working on polio eradication and staff who had participated in the smallpox eradication effort, at which the idea of creating a large network of skilled public health professionals who could provide sustained support to the polio eradication effort was advanced. The intent of this network would be to put “all hands on deck,” especially at the subnational level, to work alongside local healthcare staff to provide not only technical assistance but also much needed encouragement and enthusiasm; the STOP program would provide additional human resource capacity to countries still struggling to meet AFP surveillance standards, to ensure optimal polio vaccination coverage and support country-level partners and ministry of health (MoH) staff in the implementation of key activities [2].

The first STOP team was rapidly assembled in November 1998 and comprised 25 experienced and qualified CDC staff available for 3-month field assignments, including to remote areas with limited communication [2]. The initial STOP team received specialized training and deployed in January 1999 to 6 countries. To expand the STOP program, the CDC opened recruitment globally, beginning in 1999, providing an opportunity for public health personnel from around the world to gain unique experiences and contribute to the global polio eradication effort.

It was clear from the first STOP team mission that the ability to mobilize additional human resources and deploy them in support of MoH, WHO, and UNICEF field activities provided substantial support for the already overwhelmed polio teams on the ground. Over time, it also became apparent that this model of subnational short-term capacity support to the WHO, UNICEF, and MoHs was adaptable and could support other immunization and public health priorities beyond polio eradication. The initial terms of reference for STOP assignments were expanded in 2002 to support accelerated progress toward measles mortality reduction and development of data management systems for disease surveillance [2]. The terms of reference were further expanded in 2003, to include routine childhood immunization systems strengthening; in 2006, to support UNICEF country offices’ in their communications and social mobilization efforts; and in 2011, to support the leadership and management needs of immunization and eradication teams at the country level.

Poor program management was identified as a major limiting factor to polio eradication in Nigeria and other polio-endemic countries. GPEI’s Independent Monitoring Board indicated in 2011 that accountability, local management gaps, lack of innovation, and poorly planned and executed microplans were substantial obstacles for achieving eradication [3]. To address these obstacles, STOP 42 (2013) through STOP 44 (2014) received additional management training, specifically for team members going to Afghanistan, Chad, the Democratic Republic of the Congo, Nigeria, and Pakistan.

Although the number of polio endemic countries declined, the request for STOP support increased. Now 16 years after the program’s beginning, demand for STOP team members is at an all-time high, with requests that originate not only from polio endemic countries, but also from those at risk for poliovirus importation, those working towards achieving measles and rubella elimination goals, and those dealing with large outbreaks of vaccine-preventable diseases. MoHs and other implementing partners continue to request STOP team members to provide technical assistance in areas including disease surveillance, communications, measles and rubella elimination activities, routine immunization program strengthening, and improving immunization data quality and use. The STOP program has grown into a global network of skilled public health professionals that continues to share lessons learned and provide technical input where needed.

## ROLE OF PARTNERSHIPS IN THE STOP PROGRAM

Partnerships have been critical to the early and ongoing success of the program. By 2001, STOP team members were routinely deployed as WHO consultants, giving them temporary UN personnel status. This helped to give them recognition and status with host governments and facilitated the operational work at the country level. As UN-associated consultants, STOP team members were officially under the supervision of the host country WHO or UNICEF Representative. WHO or UNICEF in-country staff worked with STOP team members to develop work plans for their assignments, liaised with MoH counterparts to ensure the work plans reflected national priorities, and provided oversight and supervision for their work. Rotary International played a critical role by supporting the program financially and providing advocacy and endorsement for STOP teams. Many qualified Rotary members also participated in field assignments for STOP. In addition, from 2000–2012, the Canadian Public Health Association, funded by the Canadian government, collaborated with CDC to recruit and deploy French-speaking team members [2].

As UN-associated consultants, STOP team members benefited from UN administrative and logistical support while working at the operational levels; they were also subject to UN rules and requirements, including host government and UN clearances, as well as official communications with government officials. STOP team members were covered by UN security procedures that regulated in-country travel and evacuation, if required. In general, logistics (such as transportation) were provided by the host MoH, which typically received support from WHO or UNICEF. CDC has provided support for logistics in the rare situation where the Expanded Program on Immunization (EPI) country budget was severely limited.

## CHARACTERISTICS AND DURATION OF STOP ASSIGNMENTS

**Figure 1.**
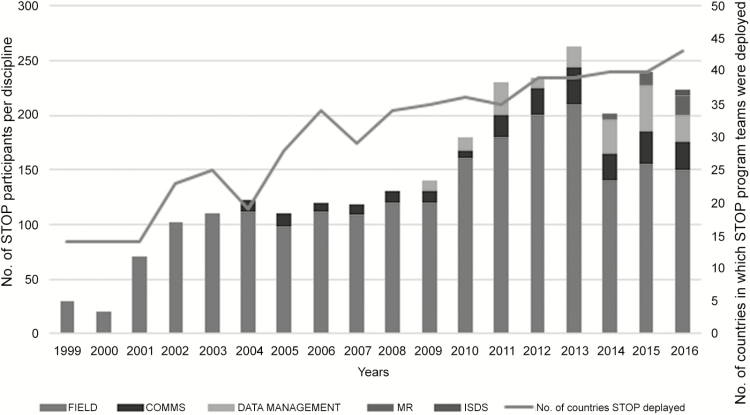
Number and composition of Stop Transmission of Polio (STOP) teams, and number of countries where STOP teams were deployed, 1999–2016. Abbreviations: COMMS, communication team members; ISDS, immunization data specialists; MR, measles and rubella team members.

**Figure 2.**
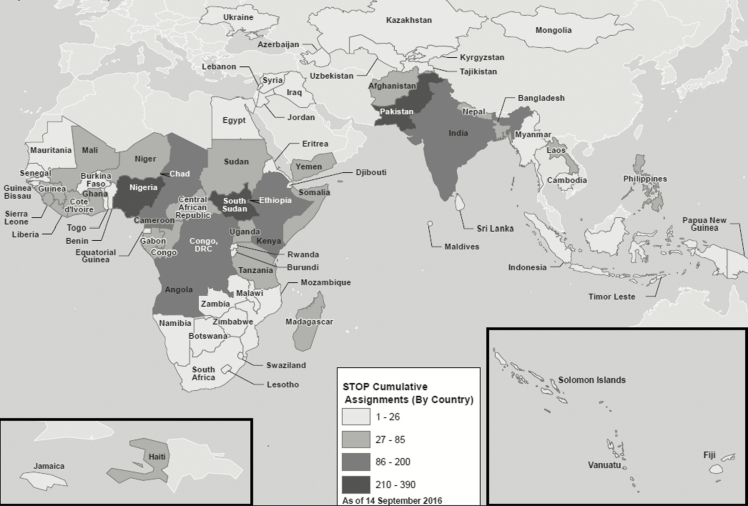
Cumulative assignments of Stop Transmission of Polio (STOP) program teams 1–48, 1999–2016.

Since its inception, the STOP program has deployed 1892 members to 48 teams. These team members have completed 3405 assignments in 75 countries (Figure 1). Twenty-four percent of deployments (n = 831) have been to countries with ongoing poliovirus transmission (including Afghanistan, Pakistan, Nigeria, and India; Figure 2). By language of assignment, 2071 have been in English, 1001 in French, 234 in Arabic, 127 in Portuguese, and 17 in Spanish speaking countries. By primary program area, 2487 assignments were related to field epidemiology, 505 to communications, 312 to data management, 96 to measles and rubella support, and 5 to a newly established Immunization Surveillance Data Specialist (ISDS) track. Assignments related to field epidemiology, measles and rubella support, and data management have been with WHO country offices, while communications assignments have been with UNICEF country offices. The STOP 48 team, which deployed in July 2016, included 216 STOP team members, including 112 field epidemiologists (28 for measles and rubella), 53 communications specialists, 18 data managers, and 5 ISDS assignments; There were 158 team members on the STOP 47 team.

From 1999–2011, the STOP program held three pre-deployment training sessions per year, and field assignments were for three months. In 2012, pre-deployment training was reduced to twice per year to allow for longer field deployments of 5 months each, for a total of 10 months in the field instead of 9 months. This change led to notable cost savings and a reduced administrative burden. Currently, team members are allowed to volunteer for up to four consecutive assignments, equivalent to almost two calendar years. This allows team members to develop comprehensive knowledge of the country and its disease surveillance and EPI activities and provides the country with a degree of continuity in staffing. STOP team members, or alumni, who return for several assignments are key to the success and continuity of the STOP program since 2006.

## NEW INITIATIVES FOR THE STOP PROGRAM

### Polio Transition With a Focus on Measles and Rubella Elimination

Since 2014, STOP has included a STOP Measles/Rubella (STOP MR) track, using the STOP platform to recruit and deploy consultants to support measles and rubella prevention, surveillance, and outbreak response activities in priority countries. Identification of priority countries and policy guidance are based on the Measles and Rubella Strategic Plan 2012–2020 [4]. The main terms of reference for STOP MR team members are to provide technical assistance and to strengthen country capacity in supplementary immunization activity (SIA) planning, implementation and evaluation; vaccine-preventable disease (VPD) surveillance systems with particular focus on measles/rubella; outbreak risk assessment and response; and strengthening EPI systems and routine immunization service delivery. STOP MR recruits mid-level public health professionals with extensive experience with field epidemiology and either routine immunization or general EPI implementation in the developing world. To date, the STOP program has deployed 42 STOP MR team members on 96 assignments in 35 countries.

### Transition of STOP Data Management Into STOP Immunization Surveillance Data Specialists

High quality data are a prerequisite to accurate information, better decision-making, and ultimately improved population health [5]. The STOP program began deploying STOP data managers in 2002; since then, STOP has deployed 168 data managers on 312 assignments in >55 different countries. In the field, STOP data managers support data cleaning, analysis, and data quality improvement activities related to AFP, measles, and rubella surveillance, as well as routine and supplemental immunization activities. STOP data managers have traditionally worked at the national level in collaboration with WHO and MoHs, providing additional capacity to manage immunization data and strengthen health information systems within their countries of assignment.

In many countries, the quality of administrative immunization coverage data is inadequate and the use of immunization data for corrective action at the district and community level is unsatisfactory [6]. Additionally, the Polio Eradication and Endgame Strategic Plan outcome indicators and data quality requirements for financial support from Gavi, the Vaccine Alliance, have introduced increasing demands for higher-quality data. In response, the STOP program modified its approach to data management to support data processes and human resource capacity at the subnational levels to improve data quality and use from the ground up [7, 8]. This approach involved the development of a new deployment role, STOP immunization and surveillance data specialists (ISDSs). ISDSs aim to improve health information systems by assessing knowledge, needs, and barriers to immunization and VPD surveillance data management, quality, and use at the lowest level, the level of primary data collection. ISDSs then provide mentorship and on-the-job training of the local staff who support these processes. A pilot of the STOP ISDS role was initiated in 2016 in Kenya. Subsequent evaluation of the impact of ISDSs will determine future incorporation of ISDSs into the routine deployment schedule of the STOP program.

## CONTRIBUTION OF STOP TO POLIO ERADICATION PROGRAMS, WORKFORCE CAPACITY, AND STRENGTHENING ROUTINE IMMUNIZATION PROGRAMS

The primary sources of information on the contribution of the STOP program include ongoing monitoring of STOP deployments and volunteers and a formal external evaluation of the STOP program. The routine monitoring system for the STOP program gathers information about implementation of program activities, team members’ experiences, and individual performance of STOP team members, and it solicits general feedback to inform program improvement. Information is collected from STOP team members via emails to STOP program management staff and through self-administered online surveys during and after training and while on assignment. STOP supervisors complete a standard UN performance evaluation for individual STOP team members after each assignment, which the STOP participant sends to WHO headquarters as part of their end-of-assignment reporting requirements.

In 2013, an external evaluation of the STOP program was sponsored by the Bill and Melinda Gates Foundation and conducted by McKinsey and Company [9]. The objective of the evaluation was to assess STOP’s impact, identify ways to optimize program implementation, and inform future strategic directions. The methods included a desk review, a survey of STOP team members and in-country stakeholders, and in-country assessments in Nigeria, the Democratic Republic of the Congo, and South Sudan. The external evaluation showed that STOP team members spent about 50% of their time training, mentoring, or coaching local workers and managers during their assignments; the other half of their time was spent filling personnel gaps or performing day-to-day operational activities (Table 1) [9].

**Table 1.** Impact Assessment Framework

	Polio Program	Other Health Programs	Public Health Organizations
**Short term** ^**a**^			
Objective	Fill capacity gaps on polio eradication activities	Fill capacity gaps on other health initiatives	…
Activities	Provide technical capacity, overcome challenges through problem solving and interpersonal skills	Routine immunization, infectious disease outbreak response (eg, cholera)	…
**Long term** ^**b**^			
Objective	Build polio-related in-country capability	Build in-country capability for RI and other health initiatives	Build a cadre of experienced public health professionals across countries through a training
Activities	On-the-job coaching for local health workers, drive or implement systemic changes (eg, new microplan templates)	On-the-job coaching for local health workers, drive or implement systemic changes (eg, new microplan templates)	…

^a^Defined as an impact between the start and end of an assignment.

^b^Defined as either an impact that continues to exist after end of an assignment or an impact over a longer period due to continuous deployment of STOP team members.

As part of ongoing program monitoring by CDC STOP team management, STOP team members self-report the most important programmatic change they felt they observed during their assignment. The following quotations (edited for spelling, grammar, and punctuation) are from STOP 47 and 48 (2016) team members on how they built workforce capacity and strengthened awareness of polio eradication activities in their country of assignment:

“[W]e worked hand in hand with the Northern Traditional Leaders Council [NTLC], whereupon we trained the traditional leaders who are supposed to move with the team during immunization plus days to support resolving noncompliance and to help the team in maximizing the immunization coverage. I worked with NTLC for almost 2 years, and we observed a very drastic fall in the numbers of noncompliance. Later, the NTLC also helped in scaling up routine immunization coverage. I was actively involved in providing the refresher and on-the-job training of frontline volunteers and helped them in linking the beneficiaries of their catchment area to the nearby health facilities so that they are able to avail the routine immunization services as and when required.” (Nigeria, 2016)

“There is improved awareness of both healthcare workers and community members on polio eradication activities, most especially on disease surveillance and routine immunization activities. [The] political commitment to Polio Eradication Initiative activities has obviously increased…as result of advocacy. [STOP team members] provided technical support and guidance to health facilities and districts to establish surveillance structures (ie, health facility surveillance teams and district rapid response teams) for continual and sustainable provision of surveillance services.” (Uganda, 2016)

“Epidemiological surveillance of AFP became more sensitive in Chad and particularly in the 4 regions [where the STOP officer worked]…. The community leaders [took] ownership of the monitoring of AFP in sites of displaced and refugee Nigerians.” (the most significant change reported by one STOP participant in Chad, 2016)

STOP MR team members are also asked to report the most important change they observed from their work. The following quotations (edited for spelling, grammar, and punctuation) from STOP 47 MR team members illustrate the results of their work:

“The reporting of measles cases from sentinel sites improved. This was as a result of training the village polio volunteers on how to do house to house case search and reporting.” (Somalia, June 2015)

“[Kenya is] preparing to introduce rubella vaccine/[measles and rubella] vaccine. As STOP MR consultant, [I] supported the MoH to analyze rubella data collected through measles case-based surveillance and present evidence for national technical advisory group to make decision for rubella vaccine introduction. [I also] supported [the] MoH to prepare applications for Gavi support for [the] MR campaign and rubella introduction into the routine immunization program. Kenya [will] introduce rubella vaccine in November 2015.” (Kenya June 2015)

“The measles/rubella risk assessment that we did helped the subnational/national staff to focus and prioritize the high-risk districts and villages. It was also useful to apply innovative strategies, such as a house-to-house strategy in early morning and late evening sessions and [vaccination of] a wider age group for polio SIAs in the response [to a circulating vaccine-derived poliovirus] outbreak in the country.” (Lao PDR, June 2016)

In terms of measurable change in program performance, South Sudan provides an additional example of the contribution of STOP team members’ activities to country-level disease surveillance and immunization programs. From 2009 to 2010, STOP support in South Sudan increased from 42 person-months to 140 person-months; during this period, the annualized non–polio-associated AFP rate increased by 70%, indicating improved surveillance system performance [9].

In addition to the immediate impact of the work of STOP team members in their assigned country, the STOP program has had longer-term beneficial effects for the GPEI and global EPI. This long-term effect is the result of the positive impact participation in STOP has had on the longer-term careers of STOP team members. The WHO and UNICEF staff working at different levels of the GPEI have long been aware that participation in STOP has launched many careers in international health for a considerable proportion of STOP team members, and the WHO and UNICEF have hired many of the STOP team members after they have completed their assignments, to strengthen their own workforce capacity.

It has been challenging to routinely collect information on and quantify the extent to which STOP team members have performed longer-term work for the WHO, UNICEF, other international agencies or national governments. However, WHO headquarters conducted a survey in 2016 to understand the level of satisfaction STOP team members had with their experience and the impact STOP participation had on their careers. In August 2015, 368 STOP team members deployed during the preceding 3 years were invited to participate in an anonymous online survey. Feedback from 321 respondents (87%) was generally very positive; 302 STOP officers (94%) noted that participation in STOP positively benefited their career in public health. There was a variety of career trajectories: 121 respondents (38%) returned to their previous job; of these, 21% of these were promoted and 32% were given additional responsibilities. A further 144 (39%) reported that, following their STOP assignment, they were able to get a job or consultancy position with the WHO, UNICEF, another UN agency, the CDC, their MoH, or a nongovernment organization.

The survey also allowed STOP team members to answer several open-ended questions, providing interesting and useful insights. The following are 3 typical responses (edited for spelling, grammar, and punctuation) from STOP team members, illustrating the effect that being part of STOP and the GPEI had on their career:

“After participating in the STOP assignment, I am now more confident in my workplace…. It has guided me to work with a more holistic approach. Moreover, the multicultural team approach helped a lot to teach me how to work in a critical condition and how to bring out the best in a team.”

“I learned from mistakes and wrong decisions. I developed managerial, communication and project management skills—which helped me to get the temporary appointment at UNICEF-Somalia and also helped to get a long-term consultancy with UNICEF-Malawi”

“It was a very exciting and good learning opportunity to work in tough and challenging conditions like in Nigeria. It helped me to develop my skills in dealing with political leaders, high government officials or traditional leaders in a diplomatic way to achieve the program objectives.”

Learning from the lessons of the STOP program, National STOP (NSTOP) programs, which are supported by the CDC and other in-country partners, have continued to enhance GPEI partnerships and built a flexible model of implementation for other health initiatives where subnational action is needed. These programs have also developed an in-country cadre of deployable public health professionals who can be called upon to address public health emergencies or implement public health initiatives of national importance. NSTOP programs have been developed in 5 counties: Nigeria, South Sudan, Uganda, Tanzania, and Pakistan. Each country has a slightly different approach and focus, but the underlying intent is to train and deploy national staff to improve disease surveillance systems and routine immunization activities at a subnational level, with a particular focus on improving AFP surveillance and, more recently, supporting measles and rubella elimination efforts.

NSTOP Nigeria began with 70 officers in 2012 and, as of 2016, has 224 officers at national, state, and local government area levels, including 1 in each of the 184 local government areas in northern Nigeria [10, 11]. Along with the STOP team members, they support the local government area to build staff capacity, update routine immunization and campaign microplans, provide supportive supervision to health facilities, improve campaign quality and surveillance, and respond to outbreaks. During the Ebola crisis in West Africa, both STOP and NSTOP members adapted their work to rapidly assist with emergency response activities.

In Uganda’s NSTOP program, which was launched in response to importations of wild poliovirus in 2008 and 2010, international STOP team members and NSTOP officers work alongside each other at the district level [12]. International STOP team members were seen as mentors and a source of support to help solve issues in the areas in which they worked. For example, during observation in a district where both NSTOP and international STOP officers were working, there was a suspected AFP case identified by the NSTOP team. They contacted the international STOP team member to consult on how to proceed. Once the case investigation form was complete, the NSTOP officers met with the international STOP officer and district staff and handed the follow-up investigation activities over to them. This shows how both the NSTOP and STOP program continue to support each other in addition to supporting district health issues while working in the same areas.

Since 2009, STOP has deployed 20 team members to South Sudan to support their EPI program. In 2015, the South Sudanese MoH, in collaboration with the CDC, the WHO, the African Field Epidemiology Network, and other partners, implemented the Human Resources Development for South Sudan EPI to build capacity within the national immunization program. The international STOP team members’ role has been to work with and mentor these 56 Sudanese public health workers in support of the national EPI program.

The NSTOP program in Pakistan was launched in March 2011 to complement polio eradication efforts outlined in the National Emergency Action Plan [13, 14]. The STOP program assisted with the first training of the NSTOP officers to help initiate the program. The current NSTOP program recruits, trains, and assigns physicians, most of whom are graduates from the National Field Epidemiology Training Program, to selected high-risk districts for assignments with durations of at least 6 months. These NSTOP officers staff district polio control rooms or provincial emergency operations centers and provide direct technical assistance to local deputy commissioners. International STOP team members may be used to backfill and support polio eradication positions typically filled by NSTOP officers, illustrating the close interaction between STOP team members and NSTOP at the district level.

The STOP program also informed the development of the Strengthening Technical Assistance for Routine Immunization Training (START) program. By implementing the Global Vaccine Action Plan recommendation to strengthen the capacity of EPI managers and service delivery staff, the START approach aims to build the capacity of district-level EPI officers in key technical and competency areas through the use of direct on-the-job training, mentoring, and follow-up visits [15]. CDC recruits START participants from a pool of applicants, who are trained for 1–2 weeks and then deployed to the field for 5.5–9 months. Since the program started in 2014, START has been implemented in Uganda, Kenya, Ethiopia, and Indonesia, deploying 12 international and 20 national staff. The majority of the international START participants were STOP program alumni. Their participation in STOP provided them with experience working with international organizations in low- and middle-income countries and conducting training for public health staff.

## CONCLUSIONS

The STOP program has contributed extensively to the GPEI, through training and deployment of skilled personnel to fill capacity gaps at the country level and through increasing the knowledge and skills of public health staff in all levels of the health system. These efforts have been focused on designing, implementing, monitoring, and strengthening disease surveillance, outbreak response, and EPI systems. STOP alumni are a network of trained and experienced public health professionals who are able to support implementation of disease eradication, elimination, and response activities globally and in their country of origin. The STOP program approach and lessons learned from its implementation have informed the development of other successful workforce capacity development initiatives around the world. The STOP program has grown remarkably in size and scope over the past 16 years, highlighting the usefulness of the approach and its applicability beyond polio eradication to building strong disease surveillance and response systems and national immunization programs. Looking toward a polio-free world, the workforce and system capacity developed through the STOP program will be an important foundation from which efforts to enhance global health security and reduce the global burden of VPDs can be based.

## Notes

***Acknowledgments.*** We thank Bob Keegan, Dr Steve Cochi, Dr Jeffrey P. Koplan, and Dr Roland Sutter for their forethought and contribution to the initiation of the STOP program.

***Financial support.*** This work was supported by the Centers of Disease Control and Prevention.

***Supplement sponsorship.*** This work is part of a supplement coordinated by the Task Force for Global Health with funding provided by The Bill and Melinda Gates Foundation and the Centers for Disease Control and Prevention.

***Potential conflicts of interest.*** All authors: No reported conflicts of interest. All authors have submitted the ICMJE Form for Disclosure of Potential Conflicts of Interest. Conflicts that the editors consider relevant to the content of the manuscript have been disclosed.
